# Acceleration of the Measurement Time of Thermopiles Using Sigma-Delta Control

**DOI:** 10.3390/s19143159

**Published:** 2019-07-18

**Authors:** Manuel Domínguez-Pumar, Eduard Pérez, Marina Ramón, Vicente Jiménez, Sandra Bermejo, Joan Pons-Nin

**Affiliations:** Micro and Nano Technologies Group, Electronic Engineering Department, Universitat Politècnica de Catalunya—BarcelonaTech, 08034 Barcelona, Spain

**Keywords:** heat flux, thermopile, sliding mode control, sigma-delta

## Abstract

This work presents a double sliding mode control designed for accelerating the measurement of heat fluxes using thermopiles. The slow transient response generated in the thermopile, when it is placed in contact with the surface to be measured, is due to the changes in the temperature distributions that this operation triggers. It is shown that under some conditions the proposed controls keep the temperature distribution of the whole system constant and that changes in the heat flux at the thermopile are almost instantaneously compensated by the controls. One-dimensional simulations and experimental results using a commercial thermopile, showing the goodness of the proposed approach, are presented. A first rigorous analysis of the control using the Sliding Mode Control and Diffusive Representation theories is also made.

## 1. Introduction

Measurement of heat fluxes on surfaces using thermopiles is an extended practice for many applications [1,2,3,4,5,6,7]. When estimating heat fluxes on surfaces by contact, though, these measurements can be slow. For instance, as shown later in this paper, very long time responses are typical in commercial thermopiles and also even with those used in micro or nanoscale calorimetry applications [8,9]. The main reason for these slow response times is that the temperature distribution in the complete system can have large stabilization times depending on the associated thermal structure [6]. The measurement process can distort the temperature in the body to be measured [5] and reaching a steady state is a slow process.

Algorithms and circuits have been proposed to obtain the response of the sensors without having to wait for their thermal stabilization. For example, in Reference [10] prediction algorithms are proposed using the thermal circuit associated to the structure. Additionally, in Reference [11] hybrid methods combining spatial and temporal measurements are used to improve the time response and the accuracy of sensors. Alternatively, it is also possible to obtain quantitative results using non-invasive methods, such as optical measurements [12,13].

This paper focuses on improving the measurement time of heat fluxes using thermopiles placed in contact with surfaces. To this end, we propose applying a sliding mode control to avoid thermal transients by keeping an almost constant temperature distribution in the thermopile. This type of control is extensively used in many applications [14,15,16] mainly due to their robustness against external disturbances and model uncertainties, such as variations of the thermal circuit of the system. In our case the control is implemented as a double-loop circuit based on thermal sigma-delta modulation [17,18]. This modulation provides an implicit analog-to-digital conversion, that makes it suitable for many sensing applications, even in the case of heterogeneous systems [19,20]. Moreover, this approach also inherits other benefits of sigma-delta converters, such as quantization noise shaping [21].

The aim of the control is to avoid the thermal transients in the thermopile. As it will be explained later in more detail, this is done by keeping constant the state variables of the thermopile, in what we call constant state operation (CSO). This implies that any external variations, such as the heat fluxes generated by the environment on the thermopile, must be compensated by the actions of the control, which constitute the output of the sensor. To achieve the CSO condition, the control has two specific objectives:

- To ensure that at the moment of placing the thermopile on the surface to be measured, the contact surfaces are at the same temperature.
- To guarantee that the spatial temperature distribution in the thermopile remains constant in time once the surfaces are in contact.

The mathematical analysis included in the paper is necessary to understand what are the capabilities and limits of the controls being proposed. For many applications, it is no longer possible to dissociate the design of the sensor from the controls being implemented during its operation. The reason is that sensor performance without using the controls would not be within the expected operational limits. For example, our research group has participated in three instruments for three NASA missions to Mars: Rover Environmental Monitoring Station, REMS (Mars Science Laboratory, 2011), TWINS (InSight, 2018) and Mars Environmental Dynamics Analyzer, MEDA (for Mars2020). Constant temperature operation has been applied in all these sensors [22,23]. The mathematical thermal modeling and analysis of the control dynamics was presented in Reference [24]. Open loop operation (constant power operation in those cases) would provide unreasonable time responses.

Additionally, controls based on sigma-delta modulation have been also proposed for MOX gas sensors [21,25] and a first mathematical analysis has been presented in Reference [26]. This means that the proposed techniques are transversal and can be applied to a number of different sensor fields. In all cases it is necessary to analyze the control dynamics from a mathematical point of view, and sensor operation and even design cannot be dissociated from this analysis. This work applies these techniques for the first time to heat flux sensors.

This paper is organized as follows. Section 2 explains the proposed control, which is analyzed in Section 3 using the Diffusive Representation and Sliding Mode Control theories. Section 4 presents and discusses 1D simulations. Finally the experimental results obtained with a commercial thermopile are presented and discussed in Section 5.

## 2. Proposed Controls

**Figure 1:**
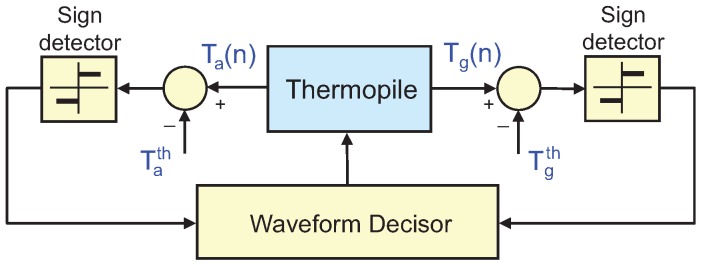
Schematic of the sigma-delta inspired controls. Details on the experimental implementation of this control strategy are provided later in Section 5.

The double control proposed is based on sigma-delta modulation. Sigma-delta modulators are discrete-time systems in which the control variables are changed periodically, with period $T_{S}$. In our case, two signals are monitored at sampling times $nT_{S}$: the voltage generated by the thermopile, proportional to the temperature difference between its top and bottom surfaces: $T_{g}\left(nT_{S}\right)=T_{\mathrm{top}}\left(nT_{S}\right)-T_{\mathrm{bottom}}\left(nT_{S}\right)$; and the average temperature, defined as $T_{a}\left(nT_{S}\right)=1/2\left(T_{\mathrm{top}}\left(nT_{S}\right)+T_{\mathrm{bottom}}\left(nT_{S}\right)\right)$, see Figure 1. Note that $T_{a}$ is simply the average temperature of the top and bottom surfaces and therefore it must not generally coincide with the average of the temperature distribution in the thermopile.

Additionally, when some current flows between the two terminals of the thermopile, two separate effects are generated [27,28]:

- Peltier effect: current flowing in one direction generates heat injection at one of the thermopile surfaces and heat extraction at the other. The heat changes sign when the current is reversed. This is the main effect in a thermopile and the heat injected/extracted at the interfaces is proportional to the value of the applied current.
- Joule effect: this is normal Joule heating and therefore it is proportional to the square of the applied current. This is a volumetric phenomenon, which happens on the bulk of the thermopile. Joule effect obviously does not produce any cooling, only heating.

**Figure 2:**
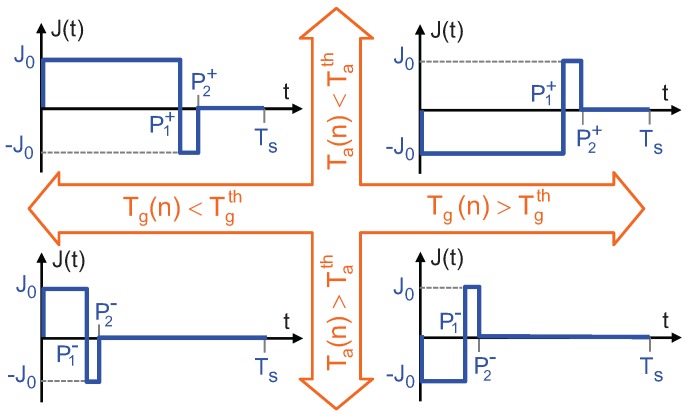
Current waveforms applied to the thermopile by the control. They imply a combined use of the Peltier and Joule effects to achieve the target temperatures $T_{g}^{\mathrm{th}}$ and $T_{a}^{\mathrm{th}}$.

According to all this, the objective of the control is to enforce the conditions $T_{g}\left(nT_{S}\right)=T_{g}^{\mathrm{th}}$ and $T_{a}\left(nT_{S}\right)=T_{a}^{\mathrm{th}}$, where $T_{g}^{\mathrm{th}}$ and $T_{a}^{\mathrm{th}}$ are previously chosen target values, by applying adequate Peltier and Joule excitations. To this effect, depending on the instantaneous value of $T_{g}\left(nT_{S}\right)$ the $T_{a}\left(nT_{S}\right)$, the control applies to the thermopile one of the current waveforms shown in Figure 2 during the next sampling period, $t\in [nT_{S},\left(n+1\right)T_{S})$.

In the next section it is shown that, if the sampling period $T_{S}$ is chosen to be much smaller that the relevant time constants in the structure, the effective excitations that these current waveforms produce are:

- a Peltier excitation, which is proportional to the average value of the current density, $\bar{J}$ and
- a Joule excitation, which is proportional to the average value of the power dissipated, that is, proportional to ${\bar{J}}^{2}$.

Then, by selecting between the two left or two right, waveforms in Figure 2 it is possible to increase or decrease the average current density $\bar{J}$ in the thermopile. Since $\bar{J}$ can be positive and negative (left or right excitations) this allows to heat or cool down the top surface (and cool or heat the bottom). Additionally, by choosing between the upper or lower waveforms, it is possible to increase or decrease the average value of the square of the current density, $\bar{J^{2}}$, which is equivalent to increase or decrease Joule heating in the thermopile.

The application of the four waveforms, governed by the double sigma-delta control loop, will allow to control the desired temperatures, $T_{a}$ and $T_{g}$.

Table 1 summarizes some of the works in the literature on the measurement of heat flux and comments on the main differences between the methods proposed in them with regard to this work. To the best knowledge of the authors, there are no works on control or feedback methods in which the thermopile itself is used as sensing and actuation. The works in References [3,29] proposed active methods for the cancellation of the heat flux across the thermopile but using external heaters. The time responses in these works are very small in some cases but most of them involve no contact (radiation heating) and/or micromechanized sensors.

## 3. Analysis of the Control

**Figure 3:**
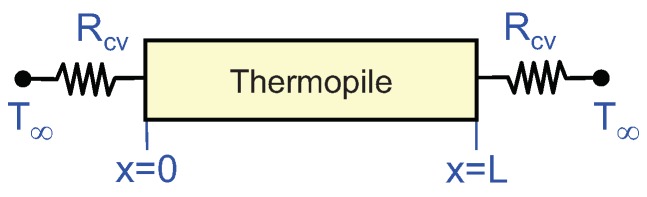
Thermopile of thickness *L* with convection losses at its surfaces. A current density J(t) is flowing inside the thermopile.

The first step towards understanding the proposed controls is to analyze their dynamics when they are applied to the thermopile before placing it on the surface. To this purpose, let us consider the case described in Figure 3, in which the thermopile is suspended on air, with negligible radiation losses. In this case all heat is lost through convection at the surfaces of the thermopile, modeled as two thermal resistances $R_{\mathrm{cv}}$.

For the sake of simplicity, let us use $T_{\infty }$, the temperature of the air, as temperature reference. In this case the heat equation is:

(1) $$C_{p}\rho_{p}\frac{\partial T}{\partial t}\left(x,t\right)=k_{p}\frac{\partial^{2}T}{\partial x^{2}}\left(x,t\right)+g_{J}\left(t\right)$$

where T(x,t), x∈[0,L], is the temperature and $C_{p}$, $\rho_{p}$ and $k_{p}$ are the thermal capacitance, the density and the thermal conductivity of the thermopile, respectively. We define:

(2) $$g_{J}\left(t\right)=\epsilon_{p}J^{2}\left(t\right)$$

as the term related to the Joule effect, being $\epsilon_{p}$ the electrical resistivity of the thermopile and J(t) the current density applied.

The boundary conditions at the thermopile surfaces x=0 and x=L are:

(3) $$\begin{array}{c} \frac{-T(t,0)}{R_{\mathrm{cv}}}=-k_{p}\frac{\partial T}{\partial x}\left(t,0^{+}\right)+g_{S}^{0}\left(t\right) \end{array}$$

(4) $$\begin{array}{c} \frac{T(t,L)}{R_{\mathrm{cv}}}=-k_{p}\frac{\partial T}{\partial x}\left(t,L^{-}\right)+g_{S}^{L}\left(t\right) \end{array}$$

where the left terms correspond to the heat losses in the surfaces of the thermopile and $g_{S}^{0}\left(t\right)=\alpha_{p}J\left(t\right)T\left(t,0^{+}\right)$ and $g_{S}^{L}\left(t\right)=\alpha_{p}J\left(t\right)T\left(t,L^{-}\right)$ are the terms related to the Peltier effect, which is proportional to the current applied J(t) [28]. There, $\alpha_{p}$ is the Seebeck coefficient of the thermopile. This formulation based on the Seebeck coefficient is similar to that used in other works [36,37]. Additionally, in our case the temperature variation in the thermopile is very small when compared to its absolute temperature and therefore we consider $g_{S}^{0}\left(t\right)≃g_{S}^{L}\left(t\right)≃g_{S}\left(t\right)$ with:

(5) $$g_{S}\left(t\right)=\alpha_{p}J\left(t\right)T_{\infty }$$

In the Laplace domain, Equations (1)–(4) become:

(6) $$\begin{array}{c} sT\left(s,x\right)=r\frac{\partial^{2}T}{\partial x^{2}}\left(s,x\right)+\frac{1}{C_{p}\rho_{p}}G_{J}\left(s\right) \end{array}$$

(7) $$\begin{array}{c} \frac{-T(s,0)}{R_{\mathrm{cv}}}=-k_{p}\frac{\partial T}{\partial x}\left(s,0^{+}\right)+G_{S}\left(s\right) \end{array}$$

(8) $$\begin{array}{c} \frac{T(s,L)}{R_{\mathrm{cv}}}=-k_{p}\frac{\partial T}{\partial x}\left(s,L^{-}\right)+G_{S}\left(s\right) \end{array}$$

where $T\left(s,x\right)=L\left\{T(t,x)\right\}$, $G_{J}\left(s\right)=L\left\{g_{J}\left(t\right)\right\}$, $G_{S}\left(s\right)=L\left\{g_{S}\left(t\right)\right\}$ and $r=k_{p}/C_{p}\rho_{p}$.

Now, defining the average temperature, $T_{a}\left(t\right):=1/2\left(T\left(t,L\right)+T\left(t,0\right)\right)$ and the gradient temperature, $T_{g}\left(t\right):=T\left(t,L\right)-T\left(t,0\right)$, together with their respective Laplace transforms, $T_{a}\left(s\right)$ and $T_{g}\left(s\right)$, it is possible to find that:

(9) $$\begin{array}{c} \begin{array}{ccc} T_{a}\left(s\right) & = & \left(T\left(s,L\right)+T\left(s,0\right)\right)/2=H_{J}^{a}\left(s\right)G_{J}\left(s\right)\\ T_{g}\left(s\right) & = & T\left(s,L\right)-T\left(s,0\right)=H_{S}^{g}\left(s\right)G_{S}\left(s\right) \end{array} \end{array}$$

with,

(10) $$\begin{array}{c} \begin{array}{ccc} H_{J}^{a}\left(s\right) & = & \frac{1}{s}\left(1-\frac{e^{\sqrt{\frac{s}{r}}L}+1}{1+e^{\sqrt{\frac{s}{r}}L}+R_{\mathrm{cv}}k_{p}\sqrt{\frac{s}{r}}\left(e^{\sqrt{\frac{s}{r}}L}-1\right)}\right)\\ H_{S}^{g}\left(s\right) & = & 2R_{\mathrm{cv}}\frac{e^{\sqrt{\frac{s}{r}}L}-1}{-1+e^{\sqrt{\frac{s}{r}}L}+R_{\mathrm{cv}}k_{p}\sqrt{\frac{s}{r}}\left(e^{\sqrt{\frac{s}{r}}L}+1\right)} \end{array} \end{array}$$

This result implies that the Joule input changes only the average temperature, whereas the Peltier input produces only variations on the gradient temperature. In both cases, the poles are simple, real and non-positive. Therefore, the impulse responses of (10) are:

(11) $$\begin{array}{cc} h_{J}^{a}\left(t\right)=\sum_{n=1}^{\infty }\eta_{n}^{a}e^{-\omega_{n}^{a}t} & t>0 \end{array}$$

(12) $$\begin{array}{cc} h_{S}^{g}\left(t\right)=\sum_{n=1}^{\infty }\eta_{n}^{g}e^{-\omega_{n}^{g}t} & t>0 \end{array}$$

where $-\omega_{n}^{a}$ and $-\omega_{n}^{g}$ are the poles of $H_{S}^{a}\left(s\right)$ and $H_{J}^{g}\left(s\right)$. Besides, $\left\{\eta_{n}^{a}\right\}$ and $\left\{\eta_{n}^{g}\right\}$ are the diffusive symbols of $h_{J}^{a}\left(t\right)=L^{-1}\left\{H_{J}^{a}\left(s\right)\right\}$ and $h_{S}^{g}\left(t\right)=L^{-1}\left\{H_{S}^{g}\left(s\right)\right\}$, respectively [38]. These diffusive symbols are the pole residues of (10). For n≫1, the pole frequencies can be approximated as:

(13) $$\omega_{n}^{a}≃\frac{4n^{2}\pi^{2}}{L^{2}}r,\;\omega_{n}^{g}≃\frac{{\left(2n-1\right)}^{2}\pi^{2}}{L^{2}}r$$

and the values of $\eta_{n}^{a}$ are positive and decreasing as $1/n^{2}$. Additionally, $\eta_{n}^{g}$ are also positive but tending to a constant value $8r/Lk_{p}$ for large *n* values.

**Figure 4:**
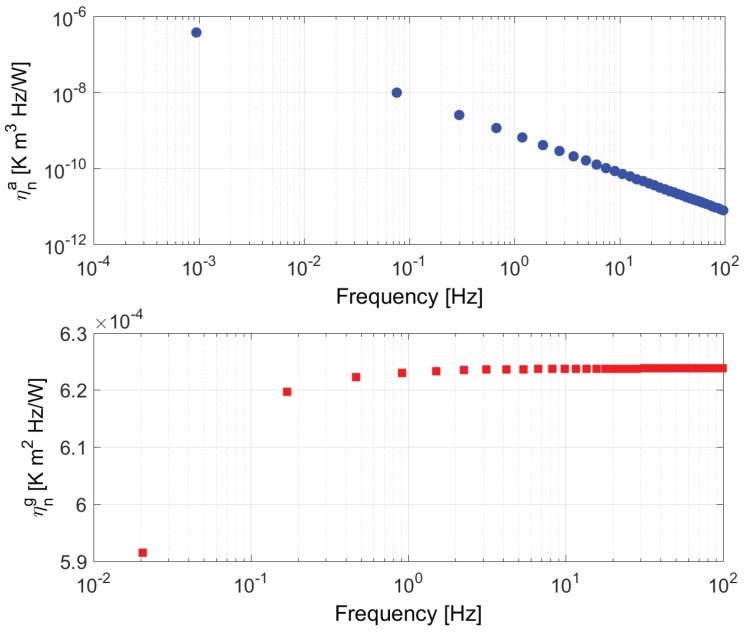
(**Top**) diffusive symbols $\eta_{n}^{a}$ corresponding to the first 40 pole frequencies $\omega_{n}^{a}$ of $H_{J}^{a}=T_{a}/G_{J}$. (**Bottom**) same for $\eta_{n}^{g}$ at $\omega_{n}^{g}$ of $H_{S}^{g}=T_{g}/G_{S}$. The frequencies of $\omega_{n}^{a}$ and $\omega_{n}^{g}$ are interspersed.

The exact values of the poles of (10) and their corresponding residues have been computed to obtain the diffusive symbols $\eta_{n}^{a}$ and $\eta_{n}^{g}$ shown in Figure 4. The thermopile parameters listed in Table 2 and $R_{\mathrm{cv}}$ = 25 mΩ have been used in the calculations. The table also includes the thermal parameters of a solid used later in the simulations. As expected, all the symbols are real and positive, with values of $\eta_{n}^{a}$ decreasing as $1/n^{2}$ with frequency. Also as expected, $\eta_{n}^{g}$ rapidly tends to $8r/Lk_{p}$ = 6.236$\times 10^{-4}$ K m${}^{2}$Hz/W.

### 3.1. Diffusive Representation of the Operators

As it has been shown, there are two impulse responses, $h_{J}^{a}\left(t\right)$ and $h_{S}^{g}\left(t\right)$, with their respective sets of poles $\left\{-\omega_{n}^{a}\right\}$ and $\{-\omega_{n}^{g}\},n>0$. In order to analyze the dynamics of the system under the infinite sampling approximation, it is convenient to use the Diffusive Representation theory [38]. Taking into account that the impulse responses present negative simple real poles, we can define a function $\psi_{n}^{a}\left(t\right)$, associated to each pole $-\omega_{n}^{a}$, in such a way that:

(14) $$\begin{array}{c} \begin{array}{ccc} {\dot{\psi }}_{n}^{a} & = & -\omega_{n}^{a}\psi_{n}^{a}\left(t\right)+g_{J}\left(t\right)\\ T_{a}\left(t\right) & = & \sum_{n>0}^{\infty }\eta_{n}^{a}\psi_{n}^{a}\left(t\right) \end{array} \end{array}$$

In the same way, for the gradient temperature, $T_{g}$, we may define functions $\psi_{n}^{g}\left(t\right)$:

(15) $$\begin{array}{c} \begin{array}{ccc} {\dot{\psi }}_{n}^{g} & = & -\omega_{n}^{g}\psi_{n}^{g}\left(t\right)+g_{S}\left(t\right)\\ T_{g}\left(t\right) & = & \sum_{n>0}^{\infty }\eta_{n}^{g}\psi_{n}^{g}\left(t\right) \end{array} \end{array}$$

with $g_{J}\left(t\right)$ and $g_{S}\left(t\right)$ defined in Equations (2) and (5). It must be noted that $\psi_{n}^{a}$ and $\psi_{n}^{g}$ are the amplitude of the excited modes of the heat equation and therefore they can be seen as state variables. This is in fact the standard approach in Diffusive Representation theory. Since the modes that both $g_{S}$ and $g_{J}$ excite are different, the state variables of both systems are independent. Joule actuation activates only symmetric temperature distributions, whereas Peltier actuation activates only antisymmetric temperature distributions.

On the other hand, the actuation signals, $g_{J}\left(t\right)$ and $g_{S}\left(t\right)$, are linked, because both depend on the applied current, J(t). For example, it is not possible to have $g_{J}\left(t\right)=0$ with $g_{S}\left(t\right)\neq 0$.

Simple Diffusive Systems—that is, systems with numerable negative single real poles—can be approximated, with arbitrary accuracy, using a finite number of poles [38]. Therefore, from here on we will consider a finite set of poles $\left\{-\omega_{n}^{a}\right\}$ and $\left\{-\omega_{n}^{g}\right\}$ for 0<n≤N. For convenience, we will assume the same cardinality in both cases.

### 3.2. InfinitE Sampling Frequency Approximation

As it has been explained in Section 2, the control applies the waveforms in Figure 2 depending on the instantaneous values of $T_{a}\left(t\right)$ and $T_{g}\left(t\right)$, which are the average and the difference, respectively, of the temperatures at the top and bottom surfaces of the thermopile. Since the control is discrete in time, control decisions are taken at multiples of the sampling period, $T_{S}$ and hence, $T_{a}$ and $T_{g}$ are discrete variables.

Following the same approach as in Reference [19], we will now analyze the control dynamics under the infinite sampling approximation. Under this approximation we consider that $T_{S}\to 0$ and that therefore the system only responds to the average value of the excitation waveforms, which have an infinitesimal duration. We may then define:

(16) $$\sigma_{a}\left(t\right):=T_{a}\left(t\right)-T_{a}^{\mathrm{th}}<0,\;\sigma_{g}\left(t\right):=T_{g}\left(t\right)-T_{g}^{\mathrm{th}}<0$$

Depending on the value of these variables, the control will apply the waveforms in Figure 2. In this case, $T_{a}\left(t\right)$ and $T_{g}\left(t\right)$ are continuous in time because of the infinite sampling approximation. Now we can define the average Joule excitation applied by the control, $u_{a}\left(t\right)=\epsilon_{p}\bar{J^{2}\left(t\right)}$, as:

(17) $$\begin{array}{c} u_{a}\left(t\right)=\left\{\begin{array}{c} \begin{array}{cccc} G_{J}^{\mathrm{on}} & = & \lim_{P_{2}^{+},T_{S}\to 0}\;\;\epsilon_{p}J_{0}^{2}\frac{P_{2}^{+}}{T_{S}} & ,\;\mathrm{if}\;\;\sigma_{a}\left(t\right)<0\\ G_{J}^{\mathrm{off}} & = & \lim_{P_{2}^{-},T_{S}\to 0}\;\;\epsilon_{p}J_{0}^{2}\frac{P_{2}^{-}}{T_{S}} & ,\;\mathrm{if}\;\;\sigma_{a}\left(t\right)>0 \end{array} \end{array}\right. \end{array}$$

and the average Peltier excitation, $u_{g}\left(t\right)=\alpha_{p}\bar{J(t)}T_{\infty }$, as:

(18) $$\begin{array}{c} u_{g}\left(t\right)=\left\{\begin{array}{c} \begin{array}{cccc} G_{+}^{\mathrm{on}} & = & \lim_{P_{1}^{+},P_{2}^{+},T_{S}\to 0}\;\;\alpha_{p}T_{\infty }\left(J_{0}\frac{P_{1}^{+}}{T_{S}}-J_{0}\frac{P_{2}^{+}-P_{1}^{+}}{T_{S}}\right) & ,\;\mathrm{if}\;\;\sigma_{g}\left(t\right)<0,\;\sigma_{a}\left(t\right)<0\\ G_{-}^{\mathrm{on}} & = & -G_{+}^{\mathrm{on}} & ,\;\mathrm{if}\;\;\sigma_{g}\left(t\right)>0,\;\sigma_{a}\left(t\right)<0\\ G_{+}^{\mathrm{off}} & = & \lim_{P_{1}^{-},P_{2}^{-},T_{S}\to 0}\;\;\alpha_{p}T_{\infty }\left(J_{0}\frac{P_{1}^{-}}{T_{S}}-J_{0}\frac{P_{2}^{-}-P_{1}^{-}}{T_{S}}\right) & ,\;\mathrm{if}\;\;\sigma_{g}\left(t\right)<0,\;\sigma_{a}\left(t\right)>0\\ G_{-}^{\mathrm{off}} & = & -G_{+}^{\mathrm{off}} & ,\;\mathrm{if}\;\;\sigma_{g}\left(t\right)>0,\;\sigma_{a}\left(t\right)>0 \end{array} \end{array}\right. \end{array}$$

Then, under the infinite sampling approximation, systems in (14) and (15) become:

(19) $$\begin{array}{c} \begin{array}{ccc} {\dot{\psi }}_{n}^{a} & = & -\omega_{n}^{a}\psi_{n}^{a}\left(t\right)+u_{a}\left(t\right)\\ T_{a}\left(t\right) & = & \sum_{n>0}^{N}\eta_{n}^{a}\psi_{n}^{a}\left(t\right) \end{array} \end{array}$$

(20) $$\begin{array}{c} \begin{array}{ccc} {\dot{\psi }}_{n}^{g} & = & -\omega_{n}^{g}\psi_{n}^{g}\left(t\right)+u_{g}\left(t\right)\\ T_{g}\left(t\right) & = & \sum_{n>0}^{N}\eta_{n}^{g}\psi_{n}^{g}\left(t\right) \end{array} \end{array}$$

In general we will consider that $G_{+}^{\mathrm{on}}>G_{+}^{\mathrm{off}}>0$ and $G_{-}^{\mathrm{on}}<G_{-}^{\mathrm{off}}<0$. In the case of the average excitation, we will consider that $G_{J}^{\mathrm{on}}>G_{J}^{\mathrm{off}}$.

### 3.3. Sliding Mode Analysis of the Average Control

Now we will analyze the dynamics of the system described in (19) and (20), with the average actuations defined in (17) and (18), which are provided by the waveforms generated by the control. As it has been mentioned before, the state variables of the system are $\psi_{n}^{a}\left(t\right)$ and $\psi_{n}^{g}\left(t\right),n=1,\cdots ,N$. Therefore we can generate two vectors, $\Psi^{a}={\left(\psi_{1}^{a},\cdots ,\psi_{N}^{a}\right)}^{T}$ and $\Psi^{g}={\left(\psi_{1}^{g},\cdots ,\psi_{N}^{g}\right)}^{T}$, representing the state of the complete system at any given time.

Since the objective of the controls is to set values to $T_{a}$ and $T_{g}$ we may speak of two control surfaces: $S_{a}\left(T_{a}^{\mathrm{th}}\right):=\left\{\Psi^{a}\in R^{N}:\sigma_{a}\left(\Psi^{a}\right)=\sum_{n}\eta_{n}^{a}\psi_{n}^{a}-T_{a}^{\mathrm{th}}=0\right\}$ and $S_{g}\left(T_{g}^{\mathrm{th}}\right):=\left\{\Psi^{g}\in R^{N}:\sigma_{g}\left(\Psi^{g}\right)=\sum_{n}\eta_{n}^{g}\psi_{n}^{g}-T_{g}^{\mathrm{th}}=0\right\}$. It is desired then that the control places $\Psi^{a}$ and $\Psi^{g}$ on the respective surfaces, which is equivalent to controlling $T_{a}$ and $T_{g}$.

#### 3.3.1. Attractive Set on $S_{a}$

The control surface for the average temperature is $S_{a}\left(T_{a}^{\mathrm{th}}\right):=\left\{\Psi^{a}\in R^{N}:\sigma_{a}\left(\Psi^{a}\right)=\sum_{n}\eta_{n}^{a}\psi_{n}^{a}-T_{a}^{\mathrm{th}}=0\right\}$, with $\Psi^{a}={\left(\psi_{1}^{a},\cdots ,\psi_{N}^{a}\right)}^{T}$. We will generally assume that $G_{J}^{\mathrm{off}}<G_{J}^{\mathrm{on}}$.

The first result is that the set:

(21) $$\Omega_{a}^{\infty }:=\left\{\Psi^{a}\in R^{N}:\psi_{n}^{a}\in \left(\frac{G_{J}^{\mathrm{off}}}{\omega_{n}^{a}},\frac{G_{J}^{\mathrm{on}}}{\omega_{n}^{a}}\right)\right\}$$

is forward invariant. This follows from having single negative poles with input $u_{a}\left(t\right)\in \left\{G_{J}^{\mathrm{off}},G_{J}^{\mathrm{on}}\right\}$.

On the other hand, we have that:

(22) $$\begin{array}{c} \dot{\sigma }=\left\{\begin{array}{c} G_{J}^{\mathrm{off}}\Gamma_{a}-\sum_{n}^{N}\eta_{n}^{a}\omega_{n}^{a}\psi_{n}^{a}\left(t\right),\;\sigma \left(\Psi^{a}\right)>0\\ G_{J}^{\mathrm{on}}\Gamma_{a}-\sum_{n}^{N}\eta_{n}^{a}\omega_{n}^{a}\psi_{n}^{a}\left(t\right),\;\sigma \left(\Psi^{a}\right)<0 \end{array}\right. \end{array}$$

where $\Gamma_{a}=\sum_{n}^{N}\eta_{n}^{a}$. Now, the intersection of the following subset of the state space:

(23) $$\Omega_{a}:=\left\{\Psi^{a}\in R^{N}:G_{J}^{\mathrm{off}}<\frac{1}{\Gamma_{a}}\sum_{n}^{N}\eta_{n}^{a}\omega_{n}^{a}\psi_{n}^{a}<G_{J}^{\mathrm{on}}\right\}$$

with the control surface $S_{a}$, that is, $\Omega_{a}\cap S_{a}$, is attractive. This is due to the fact that if $\Psi^{a}\left(t\right)\in \Omega_{a}$ and σ(t)<0 we will have $\dot{\sigma }\left(t\right)>0$, whereas if σ(t)>0 then we will have $\dot{\sigma }\left(t\right)<0$. Therefore it is $\sigma \dot{\sigma }<0$ in a neighbourhood of $\Omega_{a}\cap S_{a}$, excluding the control surface, which means that the set is attractive.

**Proposition** **1.** *If $\eta^{a}\in R_{+}^{N}\backslash \left\{0\right\}$, there is a maximal interval $I_{a}\subset R$ such that, for all $T_{a}^{\mathrm{th}}\in I_{a}$, the set $S_{a}\left(T_{a}^{\mathrm{th}}\right)\cap \Omega_{a}^{\infty }$ is an attractive non empty sliding set.*

**Proof.**  If $\eta^{a}\in R_{+}^{N}\backslash \left\{0\right\}$ we have that $\Omega_{a}^{\infty }\subset \Omega_{a}$. Then, if:

(24) $$T_{a}^{\mathrm{th}}\in I_{a}=\left(G_{J}^{\mathrm{off}}\sum_{n}^{N}\frac{\eta_{n}^{a}}{\omega_{n}^{a}},G_{J}^{\mathrm{on}}\sum_{n}^{N}\frac{\eta_{n}^{a}}{\omega_{n}^{a}}\right)$$

we have that $S_{a}\left(T_{a}^{\mathrm{th}}\right)\cap \Omega_{a}^{\infty }$ is a non empty attractive sliding set. The interval is maximal since for any $T_{a}^{\mathrm{th}}$ outside the interval (24) we have that $S_{a}\left(T_{a}^{\mathrm{th}}\right)\cap \Omega_{a}^{\infty }=\emptyset$. □

#### 3.3.2. Equivalent Control

We now may calculate the equivalent control $u_{a}^{\mathrm{eq}}\left(t\right)$:

(25) $${\dot{\sigma }}_{a}\left(t\right)=\sum_{n}^{N}\eta_{n}^{a}\left(\omega_{n}^{a}\psi_{n}^{a}\left(t\right)-u_{a}^{\mathrm{eq}}\left(t\right)\right)=0$$

and therefore:

(26) $$u_{a}^{\mathrm{eq}}\left(t\right)=\Gamma_{a}^{-1}\sum_{n}^{N}\eta_{n}^{a}\omega_{n}^{a}\psi_{n}^{a}\left(t\right)$$

The global dynamics on the control surface is then:

(27) $${\dot{\psi }}_{n}^{a}\left(t\right)+\omega_{n}^{a}\psi_{n}^{a}\left(t\right)=\frac{1}{\Gamma_{a}}\sum_{l}^{N}\eta_{l}^{a}\omega_{l}^{a}\psi_{l}^{a}\left(t\right)$$

**Proposition** **2.** *If $\eta^{a}\in R_{+}^{N}\backslash \left\{0\right\}$ and $T_{a}^{\mathrm{th}}\in I_{a}$ the system presents a unique stable equilibrium point at $S_{a}\left(T_{a}^{\mathrm{th}}\right)\cap \Omega_{a}^{\infty }$.*

**Proof.**  The system in (27) can be rewritten as $\dot{\psi }=M\psi$ with:

(28) $$M=-\mathrm{diag}\left(\omega_{1},\cdots ,\omega_{N}\right)+1_{N}\cdot \left(\eta_{1}\omega_{1},\cdots ,\eta_{N}\omega_{N}\right)/\Gamma_{a}$$

where $\mathrm{diag}(\omega_{1},\cdots ,\omega_{N})$ is the diagonal matrix with components $\omega_{n}$ and $1_{N}={\left(1,\cdots ,1\right)}^{T}\in R^{N}$. *M* is a proper Metzler matrix because its off diagonal elements are nonnegative and at least there is a nonzero entry. This means that for some ϵ>0, matrix A=M+ϵI is a positive matrix and we can then apply the Perron-Frobenius Theorem: the eigenvalue, $\lambda_{0}$, of largest absolute value of a positive square matrix, *A*, is both simple and positive and belongs to a positive eigenvector, $f\in R_{+}^{N}\backslash \left\{0\right\}$. All other eigenvalues are smaller in absolute value [39].

Matrix *M* has the same eigenvalue spectrum of *A* displaced to the left ϵ. This implies that *M* has a real simple eigenvalue $\lambda_{0}^{\prime }=\lambda_{0}-\epsilon$ such that for any other eigenvalue, λ, it is $Re\left(\lambda \right)<\lambda_{0}^{\prime }$ [39].

It is easy to check that for $\bar{x}={\left(\omega_{1}^{-1},\cdots ,\omega_{N}^{-1}\right)}^{T}$, we have $M\bar{x}=0$. Now, we will follow a procedure similar to that of [40]. If there is an eigenvalue, λ, with Re(λ)>0 then $\lambda_{0}^{\prime }\geq Re\left(\lambda \right)>0$ and therefore there is a left eigenvector, $f\in R_{+}^{N}\backslash \left\{0\right\}$, such that:

(29) $$f^{T}M\bar{x}=\lambda_{0}^{\prime }f^{T}\bar{x}=0$$

and since $\bar{x},f\in R_{+}^{N}\backslash \left\{0\right\}$ we have that $\lambda_{0}^{\prime }=0$.

The left eigenvector of the zero eigenvalue is obviously $\left(\eta_{1},\cdots ,\eta_{N}\right)$ and, therefore, the trajectory followed by the system is on the control surface $S_{a}\left(T_{a}^{\mathrm{th}}\right)$. This trajectory will tend to an asymptotic stable equilibrium on $S_{a}\left(T_{a}^{\mathrm{th}}\right)$, driven by all the remaining eigenvalues of the spectrum of *M*, since all of them have a negative real part. □

### 3.4. Sliding Mode Analysis of the Gradient Control

The control surface for the gradient temperature is $S_{g}\left(T_{g}^{\mathrm{th}}\right):=\left\{\Psi^{g}\in R^{N}:\sigma_{g}\left(\Psi^{g}\right)=\sum_{n}\eta_{n}^{g}\psi_{n}^{g}-T_{g}^{\mathrm{th}}=0\right\}$, with $\Psi^{g}={\left(\psi_{1}^{g},\cdots ,\psi_{N}^{g}\right)}^{T}$. We will generally assume that $G_{+}^{\mathrm{on}}>G_{+}^{\mathrm{off}}>0$ and $G_{-}^{\mathrm{on}}<G_{-}^{\mathrm{off}}<0$.

The gradient temperature actuation (18) can be rewritten as:

(30) $$\begin{array}{c} u_{g}=\left\{\begin{array}{c} G_{+}^{\mathrm{off}}+\left(G_{+}^{\mathrm{on}}-G_{+}^{\mathrm{off}}\right)\lambda \left(t\right),\;\mathrm{if}\;\sigma_{g}<0\\ G_{-}^{\mathrm{off}}+\left(G_{-}^{\mathrm{on}}-G_{-}^{\mathrm{off}}\right)\lambda \left(t\right),\;\mathrm{if}\;\sigma_{g}>0 \end{array}\right. \end{array}$$

where λ(t) reflects the state of the average temperature control:

(31) $$\begin{array}{c} \lambda \left(t\right)=\left\{\begin{array}{c} u_{a}^{\mathrm{eq}}\left(t\right)/\left(G_{J}^{\mathrm{on}}-G_{J}^{\mathrm{off}}\right),\;\mathrm{if}\;\sigma_{a}=0\\ \;1,\;\mathrm{if}\;\sigma_{a}<0\\ \;0,\;\mathrm{if}\;\sigma_{a}>0 \end{array}\right. \end{array}$$

**Remark** **1.** *The gradient control uses switching between $G_{+}^{\mathrm{on}}|G_{-}^{\mathrm{on}}$ or $G_{+}^{\mathrm{off}}|G_{-}^{\mathrm{off}}$ depending on the state of the average control. This means that the effective actuation of the gradient control depends on the instantaneous value of the actuation determined by the average control. This is what the terms in (31) reflect.*

*Let us now define the sets:*

(32) $$\Omega_{g}^{r}:=\left\{\Psi^{g}\in R^{N}:\psi_{n}^{g}\in \left(\frac{G_{-}^{\mathrm{off}}}{\omega_{n}^{g}},\frac{G_{+}^{\mathrm{off}}}{\omega_{n}^{g}}\right)\right\}$$

(33) $$\Omega_{g}:=\left\{\Psi^{g}\in R^{N}:G_{-}^{\mathrm{off}}<\frac{1}{\Gamma_{g}}\sum_{n}^{N}\eta_{n}^{g}\omega_{n}^{g}\psi_{n}^{g}<G_{+}^{\mathrm{off}}\right\}$$

*where $\Gamma_{g}=\sum_{n}^{N}\eta_{n}^{g}$.*

**Proposition** **3.** *If $\eta^{g}\in R_{+}^{N}\backslash \left\{0\right\}$, there is an interval $I_{g}\subset R$ such that, for all $T_{g}^{\mathrm{th}}\in I_{g}$ the set $S_{g}\left(T_{g}^{\mathrm{th}}\right)\cap \Omega_{g}^{r}$ is an attractive non empty sliding set.*

**Proof.**  If $\eta^{g}\in R_{+}^{N}\backslash \left\{0\right\}$ we have that $\Omega_{g}^{r}\subset \Omega_{g}$. Now, if:

(34) $$T_{g}^{\mathrm{th}}\in I_{g}=\left(G_{-}^{\mathrm{off}}\sum_{n}^{N}\frac{\eta_{n}^{g}}{\omega_{n}^{g}},G_{+}^{\mathrm{off}}\sum_{n}^{N}\frac{\eta_{n}^{g}}{\omega_{n}^{g}}\right)$$

then, following the same approach as in Proposition 1, $S_{g}\left(T_{g}^{\mathrm{th}}\right)\cap \Omega_{g}^{r}$ is a non empty attractive sliding set. □

#### Equivalent Control

Under the conditions of Proposition 3, we now may calculate the equivalent control $u_{g}^{\mathrm{eq}}$:

(35) $$u_{g}^{\mathrm{eq}}\left(t\right)=\Gamma_{g}^{-1}\sum_{n}^{N}\eta_{n}^{g}\omega_{n}^{g}\psi_{n}^{g}\left(t\right)$$

The dynamics on the control surface is then:

(36) $${\dot{\psi }}_{n}^{g}\left(t\right)+\omega_{n}^{g}\psi_{n}^{g}\left(t\right)=\frac{1}{\Gamma_{g}}\sum_{l}^{N}\eta_{l}^{g}\omega_{l}^{g}\psi_{l}^{g}\left(t\right)$$

**Proposition** **4.** *If $\eta^{g}\in R_{+}^{N}\backslash \left\{0\right\}$ and there is an equilibrium point of the system in $S_{g}\left(T_{g}^{\mathrm{th}}\right)\cap \Omega_{g}^{r}$, it will be unique and asymptotically stable.*

**Proof.**  It can be proven using the same approach as in Proposition 2. □

This proposition shows that, regardless of the state of the average control, it is possible to guarantee under some conditions the stability of the system in a set, $\Omega_{g}^{r}$, smaller than the one that can be reached under more favourable conditions of the average control.

### 3.5. Discussion

The results in Section 3.4 show that, regardless of the state of the average control, it is possible to guarantee under some conditions the stability of the system in a set, $\Omega_{g}^{r}$, smaller than the one that can be reached under more favourable conditions of the average control. The dynamics of the thermopile while it is in contact with the surface is more complex and subject of future work. In order to understand what is to be expected in the experimental measurements, we will proceed with simulations.

In this Section we have analyzed the dynamics of the control before the thermopile is placed on the surface. The fact that the structure is symmetrical (convection on the two sides of the thermopile) implies that the Joule excitation generates symmetrical modes of the heat equation, whereas the Peltier excitation generated antisymmetric modes. Under this approach it has been possible to obtain rigorous results with regard to the stability of the average and gradient temperature controls.

The dynamics of the thermopile while it is in contact with the surface will be more complex and subject of future work. In order to understand what is to be expected in the experimental measurements, we will proceed with simulations in the next Section.

## 4. Simulation Results

**Figure 5:**
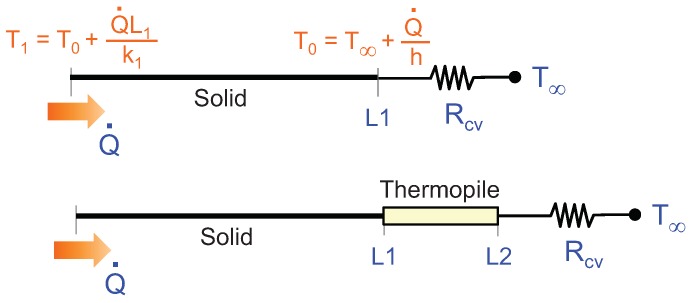
1D simulated geometry. (**Top**) domain and initial temperature distribution when no thermopile is present. (**Bottom**) domain with thermopile introduced.

To evaluate the proposed controls, discrete-time simulations with the 1D structure shown in Figure 5 have been performed. The solid corresponds to the segment $x\in [0,L_{1}]$. The heat flux $\dot{Q}$ is injected at x=0, whereas $x=L_{1}$ represents the surface on which the thermopile will be placed. We consider that this last surface dissipates heat initially through convection, $R_{\mathrm{cv}}$, before placing the thermopile. Once the thermopile is on the surface, convection takes place through the other side of the thermopile, $x=L_{2}$, see Figure 5.

The heat equation including the Joule effect is:

(37) $$\begin{array}{c} \begin{array}{cccc} C_{1}\rho_{1}\frac{\partial T}{\partial t} & = & k_{1}\frac{\partial^{2}T}{\partial x^{2}}, & x\in [0,L_{1})\\ C_{p}\rho_{p}\frac{\partial T}{\partial t} & = & k_{p}\frac{\partial^{2}T}{\partial x^{2}}+\epsilon_{p}J^{2}, & x\in [L_{1},L_{2}] \end{array} \end{array}$$

where $C_{1}$ the thermal capacitance, $\rho_{1}$ the density and $k_{1}$ the thermal conductivity of the solid.

Three different boundary conditions are applied. The first one is:

(38) $$\dot{Q}\left(t\right)=-k_{1}\frac{\partial T}{\partial x}\left(t,0\right)$$

where $\dot{Q}\left(t\right)$ is the heat flux being injected from the maximum depth in the material conforming the surface. In general we will assume that $\dot{Q}\left(t\right)$ is constant or presents very slow time-variations.

The second boundary condition is at the interface between the solid and the thermopile:

(39) $$-k_{1}\frac{\partial T}{\partial x}\left(t,L_{1}^{-}\right)=-k_{p}\frac{\partial T}{\partial x}\left(t,L_{1}^{+}\right)+\alpha_{p}J\left(t\right)T\left(t,L_{1}\right)$$

Finally, the third boundary condition is at the interface between the thermopile and the convection thermal resistance:

(40) $$-k_{p}\frac{\partial T}{\partial x}\left(t,L_{2}^{-}\right)+\alpha_{p}J\left(t\right)T\left(t,L_{2}\right)=h\left[T\left(t,L_{2}\right)-T_{\infty }\right]$$

where *h* is the convection coefficient and $T_{\infty }$ the temperature of the air at infinity. Table 2 summarizes the values of all parameters used in the simulations. The thermal parameters of the solid are those of the polypropylene used later in the experiments. The parameters of the thermopile are those of the commercial sensor used.

In the simulations, the Crank Nicolson finite difference method was used for solving the heat equations [41]. The solid and the thermopile segments were discretized in 100 points each.

### 4.1. Results and Discussion

**Figure 6:**
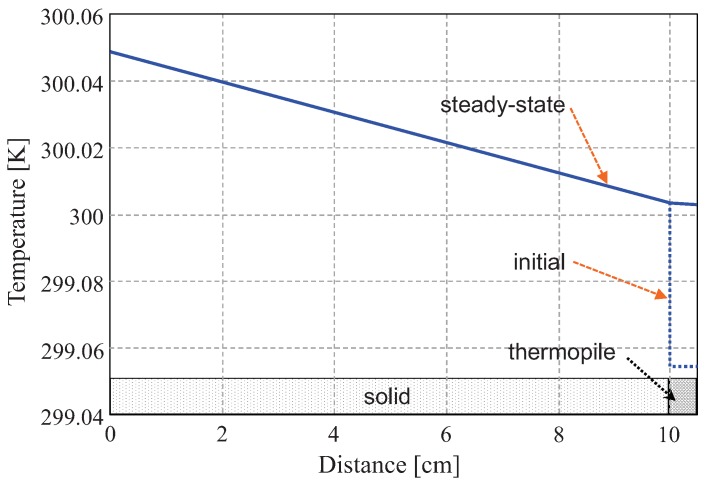
Temperature distribution in the 1D simulation domain at two different moments when no control is applied. The heat flux injected at the left of the structure is 0.1 W/m${}^{2}$. The initial temperature difference between the thermopile and the surface is 0.05 K. Steady-state is reached when the temperature distribution at the solid-thermopile interface is almost continuous.

The first simulation focuses on the evolution of the temperatures in the structure without applying any control. The initial temperature distribution is the dotted line of Figure 6. An initial difference of 0.05 K between the temperature of the surface and that of the thermopile has been introduced. The solid line of Figure 6 is the temperature distribution when, after approximately 120 min, a stable regime is reached. As it can be observed, there is no appreciable temperature discontinuity at the solid-thermopile interface. The temperature slope within the thermopile is noticeably smaller, since $k_{p}>k_{1}$.

One can conclude that the time necessary for measuring the heat flux on a surface can be generally quite large, due to the slow transients in the temperature distributions both in the surface (and underlying materials) and in the thermopile.

Now, with regard to the control and as discussed in Section 2, the objective of the controls is to set $T\left(x,t\right)=T_{\mathrm{const}}$, with $x\in \{L_{1},L_{2}\}$. Additionally, the temperature at the surface of the solid and at the contact surface of the thermopile should be the same, $T\left(L_{1}^{-}\right)=T\left(L_{1}^{+}\right)=T_{\mathrm{const}}$ at the moment of placing the thermopile in contact with the solid. To this effect, two consecutive stages can be contemplated:

- Setup: Before placing the thermopile. Controls are active.
- Operation: once the temperature of the thermopile is the same as that of the surface, the thermopile is placed on the surface.

**Figure 7:**
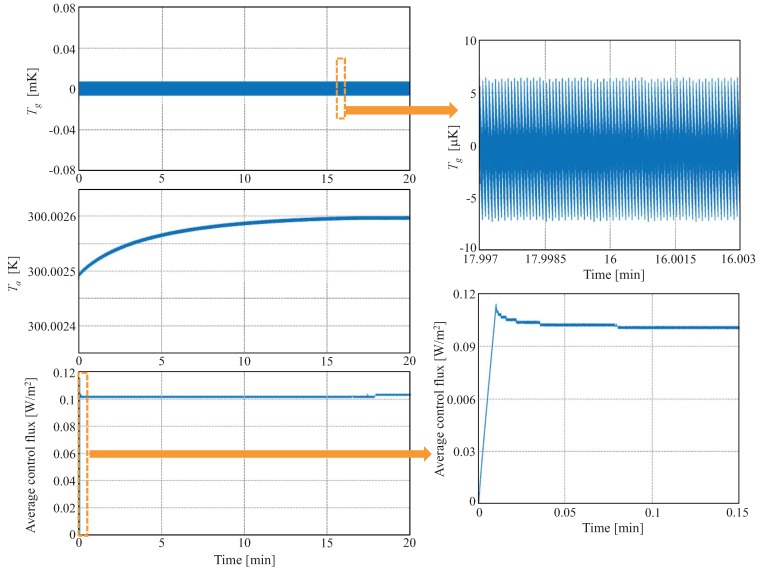
Control simulation results. At t = 0 the thermopile contacts the surface of the solid. Evolution of $T_{g}$ (**top**), $T_{a}$ (**mid**) and the average heat flux injected by the control in the thermopile (**bottom**), when the initial temperature of the thermopile is set slightly above that of the solid surface. The average heat flux is calculated as $\alpha_{p}T\left(L_{1},t\right)\bar{J}\left(t\right)$, where $\bar{J}\left(t\right)$ is the average current injected by the control. Zoom views of $T_{g}$ in steady-state and of the average heat flux during the initial transient are provided.

In the control simulations, the target average temperature $T_{a}^{\mathrm{th}}$ is set slightly above the temperature of the solid surface, therefore, near saturation. On the other hand, the target of the gradient control, $T_{g}^{\mathrm{th}}$ is set to zero. The top graph in Figure 7 shows the time evolution of $T_{g}$, being t = 0 the moment at which the thermopile contacts the surface of the solid. It can be seen that the gradient temperature fluctuates around zero with no appreciable initial transient. As it is also shown in Figure 7, the average temperature increases until approximately t = 17 min, where $T_{a}^{\mathrm{th}}$ is reached. Additionally, the bottom graph if Figure 7 shows the evolution of the gradient control flux. The time response is almost instantaneous (≈6 s).

The main function of the average temperature control is to set the thermopile at the same temperature as that of the surface to be measured. The gradient temperature control, on the other hand, is used to enforce an almost null temperature gradient in the thermopile. The simulations show that the controls allow keeping constant the temperature distributions in the whole structure: solid-thermopile. When the thermopile contacts the surface, there is a sudden change in the heat injection at the bottom of the thermopile, which is almost immediately canceled by the control.

### 4.2. Effect of Contact Resistance and Side Heat Losses

Let us now investigate the effect of two non-ideal behaviours typically present while measuring heat fluxes using thermopiles in contact with the surface to measure:

- Contact resistance: depending on the roughness of the surfaces in contact, or the applied force, a thermal contact resistance $R_{c}$ may appear between the thermopile and the surface of the solid. This effect is taken into account by modifying the boundary condition (39) as:
  (41) $$-k_{1}\frac{\partial T}{\partial x}\left(t,L_{1}^{-}\right)=\frac{T\left(t,L_{1}^{-}\right)-T\left(t,L_{1}^{+}\right)}{R_{c}}=-k_{p}\frac{\partial T}{\partial x}\left(t,L_{1}^{+}\right)+\alpha_{p}J\left(t\right)T\left(t,L_{1}^{+}\right)$$
  Any heat flux across the interface generates a temperature discontinuity drop.
- Side heat losses: this is taken into account in 1D models by adding a $-h_{L}\left(T-T_{\infty }\right)$ term, accounting for the side losses to the heat equation on the thermopile (37):
  (42) $$C_{p}\rho_{p}\frac{\partial T}{\partial t}=k_{p}\frac{\partial^{2}T}{\partial x^{2}}+\epsilon_{p}J^{2}-\mu_{p}J\frac{\partial T}{\partial x}-h_{L}\left(T-T_{\infty }\right),\;\;x\in \left[L_{1},L_{2}\right]$$

**Figure 8:**
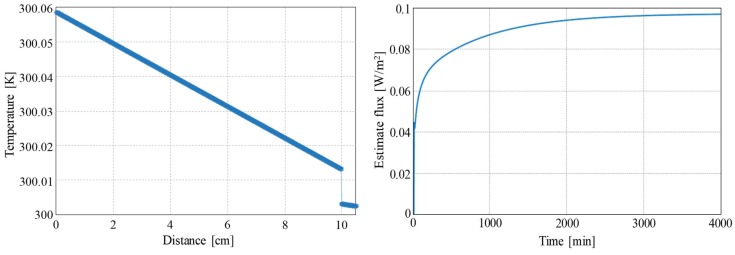
Open loop simulation results with a contact resistance of 0.1 K/(W/m${}^{2}$) and heat losses of 500 W/(m${}^{3}$K). (**Left**) final temperature distribution in the complete structure. (**Right**) Estimated heat flux in the thermopile. The heat flux injected at the left of the structure is 0.1 W/m${}^{2}$.

In the simulations we have used $R_{c}$ = 0.1 K/(W/m${}^{2}$) and $h_{L}$ = 500 W/(m${}^{3}$K). The result of simulating the structure in open loop configuration can be observed in Figure 8. The contact resistance generates a temperature drop at the interface of approximately 0.01 K. On the other hand, the asymptotic value reached by the heat flux, as estimated in the thermopile, is 0.097 W/m${}^{2}$, which is slightly below the desired value, 0.1 W/m${}^{2}$, due to the side heat losses in the thermopile.

**Figure 9:**
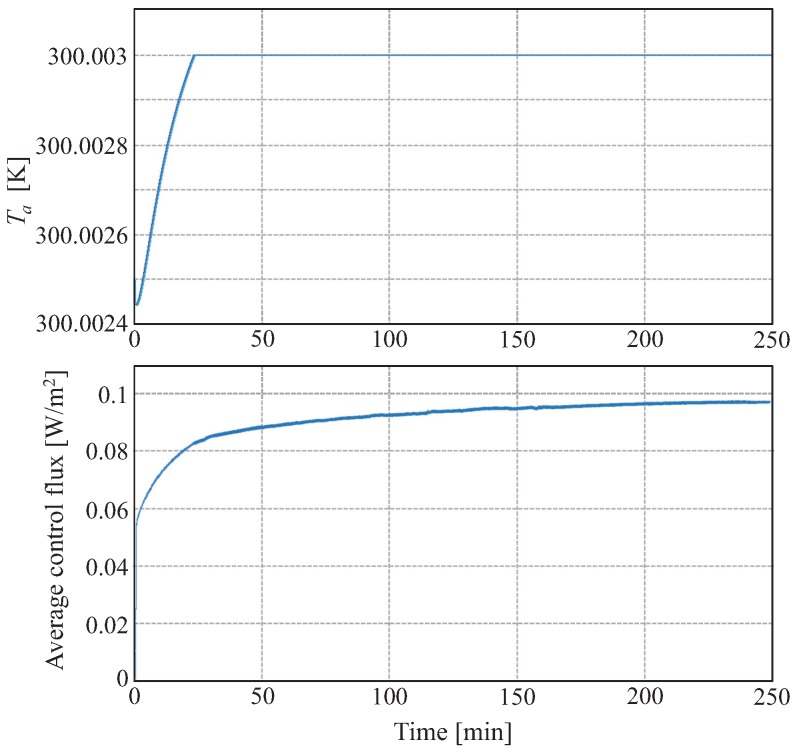
Closed loop simulation results with a contact resistance of 0.1 K/(W/m${}^{2}$) and heat losses of 500 W/(m${}^{3}$K). Evolution of the average temperature (**top**) and the estimated heat flux (**bottom**) in the thermopile. At t = 0 the thermopile contacts the surface of the solid.

Figure 9 shows the time evolution of the average temperature in the thermopile and the estimated heat flux from a simulation using the control. The main difference in the final temperature distribution is that now there is no temperature gradient in the thermopile. The dynamical response is no longer as fast as it was with no contact resistance but it is still much faster than in the open loop case.

## 5. Experimental Results

In order to test the proposed control strategy an experimental setup including a polypropylene cylinder of 15 cm diameter and 10 cm high as the solid whose surface heat flux is to be measured has been used. Two class-A accuracy PT500 resistors continuously monitor the temperatures at the top and bottom surfaces of the cylinder. The cylinder is placed on top of a Printed Circuit Board (PCB) in which a copper resistor has been drawn, which works as a constant heat source.

**Figure 10:**
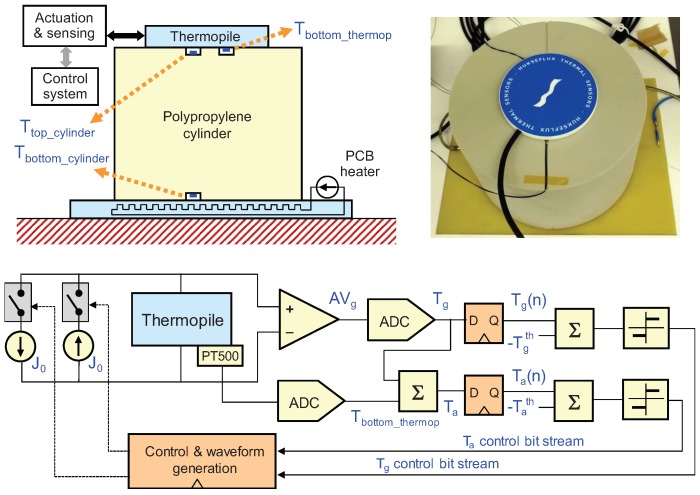
(**Top-left**) Vertical cross-section of the experimental setup; two PT500 resistors measure the temperatures at the top and at the bottom of the polypropylene cylinder; a third PT500 measures the temperature at the bottom of the thermopile, which is used to obtain $T_{a}$. (**Top-right**) Detail of the experimental setup, showing the cylinder with the PCB heater at its bottom and the thermopile at its top. (**Bottom**) implementation of the sigma-delta inspired control, including the thermopile actuation and sensing interfaces.

The thermopile used is a HFP01 commercial heat flux sensor from Hukseflux, which has a sensitivity S = 60 μV/(W/m${}^{2}$), a thermal resistivity R${}_{\mathrm{th}}$ = 0.0071 K/(W/m${}^{2}$), 8 cm${}^{2}$ of sensing area and typical response times around 180 s. A third class-A PT500 allows to monitor the temperature at the bottom-contact surface of the thermopile, as seen in Figure 10. In the experiments, the thermopile has been placed directly on top of the cylinder, with no other contact forces than its own weight. To implement the controls, only the temperature of the solid surface and the temperature at one of the surfaces of the thermopile are needed. The sensor at the bottom of the cylinder is used only to have an independent measurement to estimate the heat flux along the cylinder.

Finally, 1D simulations have been made to estimate the side heat losses in the cylinder. Assuming a convection heat coefficient of 2 W/(m${}^{2}$K) (value obtained from Reference [42] in the case of natural convection between a person and the surrounding room air), the simulations show that the difference between the heat fluxes estimated by the PT500s measurements and the one that the thermopile should be having is in the order of 10%. For the heat fluxes measured, this difference falls in the tolerance range of the PT500 resistors used.

### 5.1. Open Loop Experiment

**Figure 11:**
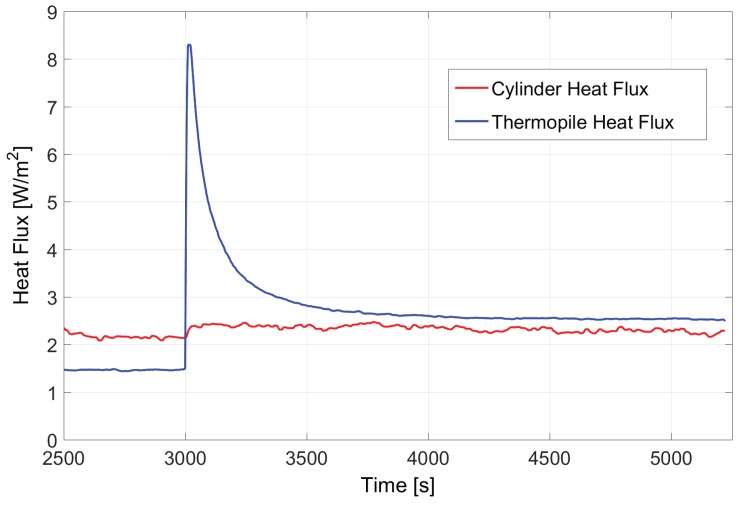
Open loop experimental results. The cylinder heat flux is monitored using the PT500 resistors at its bottom and top surfaces. At approximately t = 3000 s the thermopile is placed on top of the cylinder. The heat flux estimated by the thermopile is obtained by monitoring its open circuit voltage, ΔV(t), as Φ(t)=ΔV(t)/S, where *S* is the sensitivity of the thermopile.

Figure 11 shows the time evolution of the estimated heat fluxes in the structure in an open loop experiment. The heat flux in the thermopile is estimated from the open circuit voltage, ΔV and the sensitivity, S = 60 μV/(W/m${}^{2}$), as $\bar{\Phi }=\Delta V/S$. The heat flux in the cylinder is estimated to be constant and approximately 2.35 W/m${}^{2}$. This estimation comes from the PT500 resistors at the bottom and top surfaces of the cylinder and taking k = 0.22 W/(mK) as the thermal conductivity of polypropylene. It must be noted that more than about half the power generated at the PCB is lost to the supporting structure. The thermopile is placed in a rest position and then, at approximately t = 3000 s, it is placed on the cylinder. As it can be observed, there is a slow transient in the estimated heat flux that is stabilized after approximately 30 min. Both the heat flux estimated by the thermopile and that estimated by the PT500s placed on the cylinder are very similar and around 2.5 W/m${}^{2}$.

### 5.2. Closed Loop Experiment

In the second experiment the thermopile was under closed loop actuation with the proposed control. The current value used is $J_{0}$ = 0.42 mA/cm${}^{2}$ and the sampling frequency is $1/T_{S}$ = 1.5 KHz. Each sampling period is divided in 256 time intervals, allowing the generation of the waveforms shown in Figure 2. In this case $P_{1}^{+}$ = $215/256T_{S},P_{2}^{+}$ = $235/256T_{S}$ and $P_{1}^{-}$ = $40/256T_{S},P_{2}^{-}$ = $45/256T_{S}$.

The thermopile was placed on top of the cylinder at approximately t = 907 s and removed at t = 1430 s. The target values of the controls are $T_{g}^{\mathrm{th}}=0$ and $T_{a}^{\mathrm{th}}=T_{\mathrm{top}}$ for the whole experiment. This means that the target temperature for the top and bottom surfaces of the thermopile are equal to the temperature at the surface of the cylinder. The heat flux is calculated, as in the case of the open loop experiment, directly from the sampled output voltage of the thermopile.

**Figure 12:**
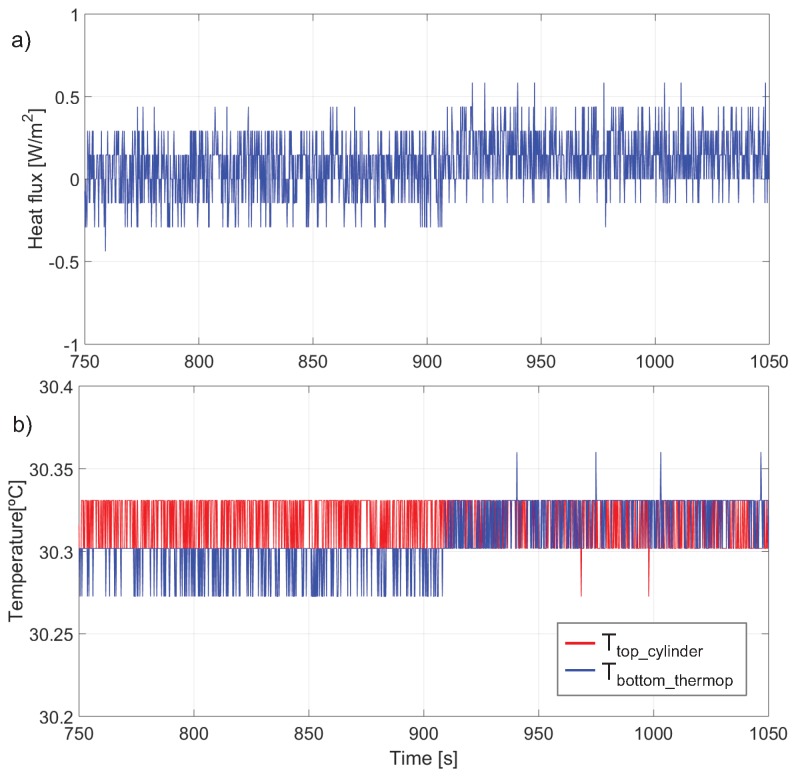
Closed loop experimental results. At approximately t = 907 s the thermopile is placed on top of the cylinder. Evolution around t = 907 s of: (**a**) the instantaneous heat flux in the thermopile (ΔV(t)/S), (**b**) the temperatures at the top of the cylinder and at the bottom of the thermopile.

Figure 12a shows the instantaneous heat flux in the thermopile near the moment the thermopile is placed on top of the cylinder, t = 907 s. The heat flux is successfully kept near zero, showing the typical chattering signal coming from a sliding control based on sigma-delta modulation. These quick variations are due to the small but fast changes in the heat flux of the thermopile generated by the application of the different current waveforms, see Figure 2.

Figure 12b shows the temperatures measured by the PT500s placed at the top surface of the cylinder and the bottom surface of the thermopile. There is a small change in the waveforms at approximately t = 907 s.

**Figure 13:**
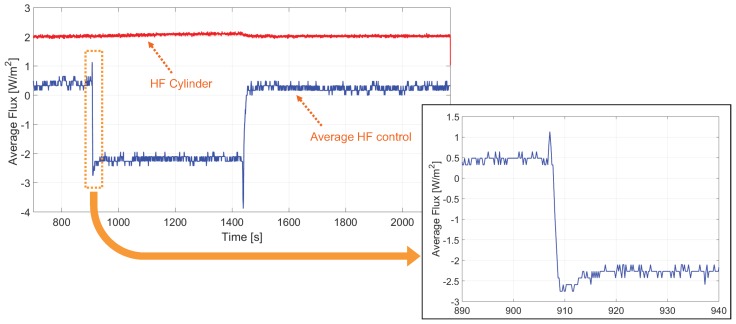
Evolution with time of the control-generated and the cylinder heat fluxes in the closed-loop experiment. The thermopile was placed on top of the cylinder at t = 907 s and removed at t = 1430 s. The cylinder heat flux monitored by the PT500s is almost constant 1.5 W/m${}^{2}$. The heat flux generated by the control compensates this heat flux change. A zoom near the first transition, t = 907 s, is also shown. Each sample is the average of 305 consecutive bits (4.9 averaged samples/s).

Figure 13 shows the evolution of the control heat flux, calculated with $\alpha_{p}=8.5\;\mathrm{mV}/K$ and $A=8\times 10^{-4}$ m${}^{2}$. In the thermopile, we define the control heat flux as negative when the current applied by the control removes heat from its bottom surface and injects heat at its top surface. Then, when at t = 907 s the thermopile is placed on top of the cylinder, it suddenly receives the heat flux coming from the cylinder. This heat flux is compensated by the control, by cooling the bottom surface of the thermopile, that is, generating a negative heat flux. Besides, before placing and after removing the thermopile the control had to compensate an average heat flux from the environment of −0.25 W/m${}^{2}$ but between t = 907 s and t = 1460 s the estimated heat flux, approximately 1.5 W/m${}^{2}$, is very close to the heat flux in the cylinder calculated from the PT500s at its top and bottom surfaces.

## 6. Conclusions

The objective of the double control proposed is to avoid slow thermal transients by achieving the CSO condition in the thermopile. This implies keeping a constant temperature distribution in the whole structure once the thermopile is placed in contact with the surface to measure. The gradient control ensures that the difference between the temperatures at the bottom-contact and top surfaces of the thermopile is constant. The average control is set to ensure that the bottom surface is at the same temperature as the surface to measure. An analysis of the dynamics of the control using the Sliding Mode Control and Diffusive Representation theories has been performed. Finally, simulations and experimental results, in which response times of a few seconds have been obtained, confirmed the goodness of the proposed approach.

## Figures and Tables

**Table 1:** Different strategies for heat flux measurement.

Ref.	Heat Flux Source	Contact	Device Type	Time Response	Comment
[11]	Jet of heated air	no	Thermopile + slug calorimeter	Reduction by 3	Needs a slug calorimeter at the back of the thermopile
[30]	Turbine blade thermal tester	no	Plug-type heat flux gauges	<50 ms	Not working when mounted on low conductivity material
[31]	Hypersonic wind tunnel	no	Schmidt–Boelter Gauge	10–15 ms	
[10]	IR radiation	no	Thermopile	<1 ms	Needs estimation of the pixel thermal time constant
[12,13]	Thermal imaging of air convection	no	Lasers + CCD CMOS	Convection stabilization: 50–75 min	Needs high heat flux levels
[32]	Fluid flow	yes	Thermopile	100 ms	The required thermal modulation can only be made with microfluidics or MEMS devices
[33]	Body heat flux	yes	Thermopile	Depends on thermostat temperature	The required mathematical modeling requires a priori knowledge
[34]	Incident radiation	no	Thermopile	<20 s	Passive and or active (using water flow) cooling is used to keep constant temperature of the sensors
[35]	Body heat flux	yes	Temperature difference between materials	Dozens of minutes	Wait for steady state response
[3,29]	Fluid flow	yes	Thermocouples	<25 s	Heat flow cancellation across thermopile is accomplished using external heaters

**Table 2:** Parameter values used in the theory and simulations.

**Solid**	$L_{1}$ (cm)	$k_{1}$ (W/mK)	$C_{1}$ (J/Kg K)	$\rho_{1}$ (Kg/m${}^{3}$)
	10	0.22	1920	910
**Thermopile**	$L_{2}-L_{1}$ (cm)	$k_{p}$ (W/mK)	$C_{p}$ (J/Kg K)	$\rho_{p}$ (Kg/m${}^{3}$)
	0.5	0.76	950	2700
	$\alpha_{p}$ (V/K)	$\mu_{p}$ (V/K)	$\epsilon_{p}$ (Ωm)	$T_{\infty }$ (K)
	8.5 × 10${}^{-3}$	45 × 10${}^{-6}$	1.00	300
**Air**	h (W/m${}^{2}$K)	$T_{\infty }$ (K)		
	40	300		

## References

[1] Xu D., Wang Y., Xiong B., Li T. (2017). MEMS-based thermoelectric infrared sensors: A review. Front. Mech. Eng..

[2] Hettegger M., Streibl B., Biro O., Neudorfer H. (2012). Measurements and Simulations of the Convective Heat Transfer Coefficients on the End Windings of an Electrical Machine. IEEE Trans. Ind. Electron..

[3] Dijkstra M., Lammerink T., de Boer M., Berenschot E., Wiegerink R., Elwenspoek M. (2014). Thermal Flow-Sensor Drift Reduction by Thermopile Voltage Cancellation via Power Feedback Control. IEEE J. Microelectromech. Syst..

[4] Has U., Wassilew D. (1999). Temperature control for food in pots on cooking hobs. IEEE Trans. Ind. Electron..

[5] Singh S., Yadav M., Khandekar S. (2017). Measurement issues associated with surface mounting of thermopile heat flux sensors. Appl. Therm. Eng..

[6] Nam S., Lee Y., Lee S. (2016). Thermal characterization of a bio sample using a heat flux sensor-based multipurpose AC microcalorimeter. Appl. Therm. Eng..

[7] Lu Y., Wang Y., Ren T. (2013). Using Late Time Data Improves the Heat-Pulse Method for Estimating Soil Thermal Properties with the Pulsed Infinite Line Source Theory. Vadose Zone J..

[8] Wolf A., Hartmann T., Bertolini M., Schemberg J., Grodrian A., Lemke K., Förster T., Kessler E., Hänschke F., Mertens F. (2015). Toward high-throughput chip calorimetry by use of segmented-flow technology. Thermochim. Acta.

[9] Krenger R., Lehnert T., Gijs M. (2018). Dynamic microfluidic nanocalorimetry system for measuring Caenorhabditis elegans metabolic heat. Lab Chip.

[10] Grönroos M., Nevalainen T., Paasio A. (2018). Implementation of a Fast and Low-Power Thermopile Readout Circuit Arrangement for Array Processors. IEEE Trans. Circuits Syst. II Express Briefs.

[11] Hubble D., Diller T. (2009). A Hybrid Method for Measuring Heat Flux. J. Heat Transf..

[12] Kumar V., Shakher C. (2015). Study of heat dissipation process from heat sink using lensless Fourier transform digital holographic interferometry. Appl. Opt..

[13] Sajith V., Sobhan C. (2012). Characterization of Heat Dissipation from a Microprocessor Chip Using Digital Interferometry. IEEE Trans. Compon. Packag. Manuf. Technol..

[14] Utkin V. (1992). Sliding Modes in Optimization and Control Problems.

[15] Chen F., Li X., Kraft M. (2016). Electromechanical Sigma-Delta Modulators (ΣΔM) Force Feedback Interfaces for Capacitive MEMS Inertial Sensors: A Review. IEEE Sens. J..

[16] Chen F., Zhou W., Zou H., Kraft M., Li X. Self-clocked dual-resonator micromachined Lorentz force magnetometer based on electromechanical sigma-delta modulation. Proceedings of the 2018 IEEE Micro Electro Mechanical Systems (MEMS).

[17] Norsworthy S., Schreier R., Temes G. (1997). Delta-Sigma Data Converters: Theory, Design, and Simulation.

[18] Chen J., Hwang Y., Jheng C., Ku Y., Yu C. (2018). A Low-Electromagnetic-Interference Buck Converter with Continuous-Time Delta-Sigma-Modulation and Burst-Mode Techniques. IEEE Trans. Ind. Electron..

[19] Dominguez-Pumar M., Gorreta S., Pons-Nin J. (2016). Sliding Mode Analysis of the Dynamics of Sigma-Delta Controls of Dielectric Charging. IEEE Trans. Ind. Electron..

[20] Jeffrey M., Kafanas G., Simpson D. (2018). Jitter in Piecewise-Smooth Dynamical Systems with Intersecting Discontinuity Surfaces. Int. J. Bifurc. Chaos.

[21] Kowalski L., Pons-Nin J., Navarrete E., Llobet E., Domínguez M. (2018). Using a Second Order Sigma-Delta Control to Improve the Performance of Metal-Oxide Gas Sensors. Sensors.

[22] Domínguez M., Jiménez V., Ricart J., Kowalski L., Torres J., Navarro S., Romeral J., Castañer L. (2008). A hot film anemometer for the Martian Atmosphere. Planet. Space Sci..

[23] Gómez-Elvira J., Armiens C., Castañer L., Domínguez M., Genzer M., Gómez F., Haberle R., Harri A.M., Jiménez V., Kahanpää H. (2012). REMS: The Environmental Sensor Suite for the Mars Science Laboratory Rover. Space Sci. Rev..

[24] Atienza M., Kowalski L., Gorreta S., Jiménez V., Domínguez-Pumar M. (2018). Thermal dynamics modeling of a 3D wind sensor based on hot thin film anemometry. Sens. Actuators A Phys..

[25] Dominguez-Pumar M., Kowalski L., Calavia R., Llobet E. (2016). Smart control of chemical gas sensors for the reduction of their time response. Sens. Actuators B Chem..

[26] Monge-Villora O., Dominguez-Pumar M., Olm J. (2018). Analysis of the dynamics of an active control of the surface potential in metal oxide gas sensors. Comput. Chem. Eng..

[27] Tritt T. (2002). Thermoelectric Materials: Principles, Structure, Properties, and Applications. Encyclopedia of Materials: Science and Technology.

[28] Lee H. (2017). Thermoelectrics: Design and Materials.

[29] Dijkstra M., Lammerink T.S.J., de Boer M.J., Berenschot J.W., Wiegerink R.J., Elwenspoek M. Low-drift flow sensor with zero-offset thermopile-based power feedback. Proceedings of the 2008 Symposium on Design, Test, Integration and Packaging of MEMS/MOEMS.

[30] Liebert C.H. Miniature High Temperature Plug-Type Heat Flux Gauges. Proceedings of the 38th International Instrumentation Symposium.

[31] Kidd C.T., Adams J.C. (2001). Fast-Response Heat-Flux Sensor for Measurement Commonality in Hypersonic Wind Tunnels. J. Spacecr. Rockets.

[32] Nam S.K., Kim J.K., Cho S.C., Lee S.K. (2010). Design and Characterization of a High Resolution Microfluidic Heat Flux Sensor with Thermal Modulation. Sensors.

[33] Socorro F., Rodríguez de Rivera P., Rodríguez de Rivera M., Rodríguez de Rivera M. (2017). Mathematical Model for Localised and Surface Heat Flux of the Human Body Obtained from Measurements Performed with a Calorimetry Minisensor. Sensors.

[34] Pullins C.A., Diller T.E. (2012). Direct Measurement of Hot-Wall Heat Flux. J. Thermophys. Heat Transf..

[35] Cousin P., Gehin C., Poujaud J., Noury N. A portable heat flux sensor. Proceedings of the 36th Annual International Conference of the IEEE Engineering in Medicine and Biology Society.

[36] Thonhauser T., Mahan G., Zikatanov L., Roe J. (2004). Improved supercooling in transient thermoelectrics. Appl. Phys. Lett..

[37] Fong E., Lam T., Fischer W., Yuan S. (2019). Analytical Approach for Study of Transient Performance of Thermoelectric Coolers. J. Thermophys. Heat Transf..

[38] Laudebat L., Bidan P., Montseny G. (2004). Modeling and optimal identification of pseudodifferential electrical dynamics by means of diffusive representation-part I: Modeling. IEEE Trans. Circuits Syst. I Regul. Pap..

[39] MacCluer C. (2000). The Many Proofs and Applications of Perron’s Theorem. SIAM Rev..

[40] De Leenheer P., Aeyels D. (2001). Stabilization of positive linear systems. Syst. Control Lett..

[41] Nguyen N., Pochiraju K. (2013). Behavior of thermoelectric generators exposed to transient heat sources. Appl. Therm. Eng..

[42] Incropera F., Dewitt D., Bergman T., Lavine A. (2007). Fundamentals of Heat and Mass Transfer.

